# Prevalence of suspected developmental delays in early infancy: results from a regional population-based longitudinal study

**DOI:** 10.1186/s12887-015-0528-z

**Published:** 2015-12-17

**Authors:** Lisbeth Valla, Tore Wentzel-Larsen, Dag Hofoss, Kari Slinning

**Affiliations:** National Network for Infant Mental Health in Norway, Center for Child and Adolescent Mental Health, Eastern and Southern Norway, Oslo, Norway; Center for Child and Adolescent Mental Health, Eastern and Southern Norway, Oslo, Norway; Norwegian Center for Violence and Traumatic Stress Studies, Oslo, Norway; Institute of Health and Society, Faculty of Medicine, University of Oslo, Oslo, Norway; Department of Psychology, University of Oslo, Oslo, Norway

**Keywords:** Ages and stages questionnaire, Suspected developmental delay, Prevalence, Infants, Screening

## Abstract

**Background:**

Prevalence estimates on suspected developmental delays (SDD) in young infants are scarce and a necessary first step for planning an early intervention. We investigated the prevalence of SDD at 4, 6 and 12 months, in addition to associations of SDD with gender, prematurity and maternal education.

**Methods:**

This study is based on a Norwegian longitudinal sample of 1555 infants and their parents attending well-baby clinics for regular health check-ups. Moreover, parents completed the Norwegian translation of the Ages and Stages Questionnaires (ASQ) prior to the check-up, with a corrected gestational age being used to determine the time of administration for preterm infants. Scores ≤ the established cut-offs in one or more of the five development areas: communication, gross motor, fine motor, problem solving and personal-social, which defined SDD for an infant were reported. Chi-square tests were performed for associations between the selected factors and SDD.

**Results:**

According to established Norwegian cut-off points, the overall prevalence of SDD in one or more areas was 7.0 % (10.3 % US cut-off) at 4 months, 5.7 % (12.3 % US cut-off) at 6 months and 6.1 % (10.3 % US cut-off) at 12 months. The highest prevalence of SDD was in the gross motor area at all three time points. A gestational age of < 37 weeks revealed a significant association with the communication SDD at 4 months, and with the fine motor and personal social SDD at 6 months. Gender was significantly associated with the fine motor and problem solving SDD at 4 months and personal- social SDD at 6 months: as more boys than girls were delayed. No significant associations were found between maternal education and the five developmental areas of the ASQ.

**Conclusion:**

Our findings indicate prevalence rates of SDD between 5.7 and 7.0 % in Norwegian infants between 4 and 12 months of age based on the Norwegian ASQ cut-off points (10.3–12.3 %, US cut-off points). During the first year of life, delay is most frequent within the gross motor area. Special attention should be paid to infants born prematurely, as well as to boys. Separate norms for boys and girls should be considered for the ASQ.

**Electronic supplementary material:**

The online version of this article (doi:10.1186/s12887-015-0528-z) contains supplementary material, which is available to authorized users.

## Background

Many studies have described the negative impacts of developmental delays in children, including emotional, behavioural and health problems later in life [1, 2], difficulties in parental child care and the parent-child relationship [3, 4], educational achievement [4, 5] and economic impacts on the families and societies [6–10]. Early identification and intervention for developmental delays cause an improvement in the successful functioning of affected children [11–14]. Research has demonstrated that intervention programmes are cost-effective and may have lifelong benefits, and also that developmental attainment is maximized when intervention is started early [11–15]. A necessary first step in order to plan for early intervention is estimation of prevalence of developmental delay and knowledge about the types of delays.

Estimates from the World Health Organization (WHO) indicate that 5 % of the world’s children under 15 years of age have some type of moderate to severe disability [16]. In the United States developmental disabilities occur in 15 % of children from 3 to 17 years of age [17]. In Norway and Scandinavia, data on the developmental status on children is scarce and the few published studies of children below school age show divergent results, varying from 6.3 % to 33 % [18, 19]. Developmental screening programmes have been shown to improve the identification and referral of children who have possible delays [20–22]. One of the validated screening tools recommended by the American Academy of Pediatrics is the Ages and Stages Questionnaires (ASQ) [23], which is a parent-completed tool for identifying infants and young children at risk for developmental delays. To date, no such recommendation exists in Norway and the Scandinavian countries, however, a Norwegian translation of the ASQ 2nd edition with a Norwegian reference (N ref.) sample has been available since 2003 [18]. The public health system in Norway provides free medical, mental and dental services for all children and youth from 0–18, and close to 100 % of parents with young infants come regularly to local well-baby clinics from birth and up to 5 years of age for weight control, vaccination and a developmental check-up of their infant [24]. Check-ups and developmental monitoring in the well-baby clinic are primarily done by public health nurses and a general practitioner (GP). Both the monitoring and check-ups are essentially based on clinical judgement and not on the use of standardized screening or assessment tools. No official definition exists regarding who is eligible for early intervention at the primary care level; thus the health providers’ clinical judgement, in combination with parent concerns, are the primary drivers for this decision.

If specialist services are needed, the local GP has to make a formal referral and get written consent from the child’s parents. Even so, there is a growing amount of interest for screening tools for developmental delay by professionals in primary care. Without accurate prevalence data based on standardized instruments, it is difficult for primary health care to adequately plan the necessary assessment and intervention responses. A lack of estimates on developmental delays among infants and children also has provided an unclear picture for policy-makers for a decision to provide early intervention services, as well as for planning and estimating the costs of early social, medical and educational intervention programmes. Hence, there is a pressing need for empirical data on knowledge about the prevalence of children at risk of developmental delay in Norway. This study seeks to contribute to building a more comprehensive picture of young infants’ developmental status.

Child development is influenced by bio-medical and socio-cultural factors that are in a continuous interaction [25]. A number of risk factors associated with an increased risk for developmental delay have been identified, including child gender, gestational age and the mothers’ educational level. Predictors of developmental delays can be useful in estimating the potential for delayed development in the population, in addition to providing an opportunity to create environments that support optimal development. The aim of this study was to estimate prevalence rates of SDD among infants at 4, 6 and 12 months of age based on parent-completed ASQ, and to investigate associations of SDD with gender, gestational age < 37 weeks and maternal education.

## Methods

### Participants

This study is based on a Norwegian population-based prospective cohort study on children’s early development from birth to two years of age. Recruitment took place between May 2011 and May 2012, and the participants were recruited from all existing well-baby clinics in five municipalities, both in urban and rural areas. Every expectant or new mother who came to these clinics was invited to participate in the study by a mid-wife or a public health nurse at their first consultation, either during pregnancy or soon after birth. The study had no specific exclusion criteria since the well-baby clinics offer services to all families with children below 5 years who live in the municipality. Mothers of 1555 children and their partners consented to participate (88.5 %). In > 95 % of cases, it was the mother who completed the ASQ. Mothers who did not consent to participate in the study differed from participating mothers in terms of having a lower educational level (*p* < 0.001) and higher proportion of non-Scandinavian speaking mothers (*p* < 0.001). No significant differences were found in gender, birth weight and gestational age between participating and non-participating children.

The current study reports on infant developmental status at 4, 6 and 12 months. The number of infants with a parent-completed ASQ form for each assessment point varied (4 months: *n* = 1244, 6 months: *n* = 1192 and 12 months: *n* = 832). The background characteristics of the study population from each assessment point are summarized in Table 1. One of the municipalities with four well-baby clinics did not collect ASQ information on the children at the 12-months consultation due to time restrictions at this particular consultation, which is the primary reason for the low number of ASQ data at 12 months.

**Table 1:** Characteristics of the study population

	Children					
	4 months		6 months		12 months	
	*n* = 1244	%	*n* = 1192	%	*n* = 832	%
Gender^a^						
Girls	562	48.6	525	47.6	372	48.5
Boys	594	51.4	579	52.4	395	51.5
Gestational age < 37 week^b^	66	5.9	59	5.5	42	5.8
Birth Weight < 2500 gr^c^	48	4.2	40	3.6	21	2.7
	Mothers					
	4 months		6 months		12 months	
	*n* = 1244	%	*n* = 1192	%	*n* = 832	%
Married or cohabitants^d^	1108	95.7	1060	95.8	746	96.5
Higher education^e,f^	726	64.0	702	64.9	524	70.0

^a^Gender has 1156 valid values at 4 months, 1104 at 6 months and 767 at 12 months, when at least one ASQ area is validity answered

^b^Gestation age 4 months (*n* = 1113) : range 26- 42 weeks, mean 39.5 weeks. Gestation age 6 months (*n* = 1065): range = 27-42 weeks, mean 39.5 weeks. Gestation age 12 months (n = 727): range 27-42 weeks, mean 39.5 weeks, when at least one ASQ question is validity answered

^c^Birth Weight 4 months (*n* = 1156): range = 772-5180 gr, mean = 3530 gr, 6 months (*n* = 1103): range = 966- 5180 gr, mean = 3547 gr, 12 months (*n* = 766): range = 966-5040 gr, mean = 3566 gr, when at least one ASQ question is validity answered

^d^Marital status has 1158 valid values at 4 months, 1106 at 6 months and 769 at 12 months,when at least one ASQ question is validity answered

^e^Higher education: Had qualified from, or studied at the university or college

^f^Education has 1134 valid values at 4 mo, 1108 at 6 mo and 749 at 12 mo, when at least one ASQ area is validity answered

### Procedure

The public health nurse or midwife provided written and oral information about the study to the parents based on procedures approved by the Norwegian Regional Committee for Medical and Health Ethics, and parents who volunteered gave their written consent to participate. On enrolment or at the first check-up after birth, background information data such as educational level, civil status, child’s gender, gestational age, and birth weight were collected and recorded. The ASQ was mailed to the participants’ home address two weeks before the 4, 6 or 12 months well-baby clinic visit. For infants born prematurely, the corrected age was used when completing the questionnaires [23]. The parents brought with them the completed ASQ to the scheduled appointment and the information on the ASQ was included as part of the overall clinical evaluation process that took place together with the parents and their child. All parents with ASQ screen positive infants were offered further evaluations of their child within two weeks, as well as referrals to specialist care in severe cases.

### Measures

The infants’ development was assessed by the Norwegian version of the Ages and Stages Questionnaire, 2nd edition [18, 23], at 4, 6 and 12 month. The ASQ is a parent-completed, developmental screening instrument, and consists of 21 age-specific questionnaires intended for use from the age of 2 months to 60 months [26]. Each questionnaire in the ASQ consists of 30 items covering five areas: communication, gross motor, fine motor, problem solving, and personal-social. Sum scores for the 6 ASQ areas were computed when all ASQ items were valid. Parents were asked to evaluate whether their child had achieved a milestone (“yes”, 10 points), had partly achieved a milestone (“sometimes”, 5 points) or had not yet achieved a milestone (“not yet”, 0 points). Each area total score is compared to a cut-off score. A child who obtains one or more area scores at or below the established cut-off levels is per definition suspected of developmental delay and should be referred for further evaluation. According to the US manual for ASQ, children who score 2 SD or more below average are considered of a suspected delay [26]. The ASQ may be used in a variety of settings (mail, online, telephone, interview, home visit, office of child care or physician) and both as parent reported and reported by health professionals [26]. The original ASQ has been proven to be a valid and reliable screening test, even in its translated and culturally adapted versions in several studies in different populations of children [27–32]. According to the Norwegian manual, the cut-off is primarily based on the 2nd percentile [18]. A construct validation study based on the N ref.sample confirmed the Norwegian ASQ version as an effective diagnostic tool of developmental delay [28]. Because no Norwegian concurrent validation study has been published, we decided to present prevalence data based on both the Norwegian and US cut-off scores.

### Data analysis

The summary of the data is presented as frequencies and percentages. The associations of SDD at 4, 6 and 12 months with gender, a gestational age of < 37 weeks and maternal education were investigated by chi-square tests. The level of significance was set at 0.05, and the data were analysed using the Statistical Package for Social Science (SPSS) software package version 22 (IBM Corp., Armonk, NY).

## Results

Complete ASQ scores were available for 1244 of the participants at 4 months, 1192 at 6 months and 832 at 12 months. The characteristics of the participating children and their mothers at 4, 6 and 12 months are presented in Table 1. The mothers’ age at the three time point ranged from 17–44, with a mean age of 30.

Table 2 shows the proportion of infants with SDD according to the Norwegian and US cut-off points in the five developmental ASQ areas at 4, 6 and 12 months.

**Table 2:** The percentage of infants scoring at or below the Norwegian (N ref.) and US cut-off values at 4, 6 and 12 months

	4 months		6 months		12 months	
	*N* ref.	US	*N* ref.	US	*N* ref.	US
	Cut-off	Cut-off	Cut-off	Cut-off	Cut-off	Cut-off
Communication	1.4 %	1.4 %	0.6 %	0.6 %	0.7 %	2.5 %
Gross motor	2.6 %	2.6 %	2.3 %	5.9 %	3.6 %	8.7 %
Fine motor	1.9 %	4.6 %	1.8 %	1.8 %	0.4 %	0.4 %
Problem- solving	2.5 %	2.5 %	1.0 %	0.2 %	1.8 %	1.8 %
Personal- social	1.1 %	3.6 %	1.5 %	3.7 %	0.5 %	1.4 %
Scoring at or below at least one area	7.0 %	10.3 %	5.7 %	10.3 %	6.1 %	12.3 %

Recommended cut-off scores in the US (Mean – 2 SD)and in the Norwegian manual (primarily based on the 2 percentile) in the areas of Ages and Stages Questionnaire, see Additional file 1

As shown in Table 2, the overall prevalence of infants scoring at or below the cut-off points of at least one developmental area according to the Norwegian cut-off points was 7.0 % at 4 months (10.3 % according to the US cut-off), 5.7 % at 6 months (10.3 % by the US cut-off), and 6.1 % at 12 months (12.3 % by the US cut-off). The percentage of infants with SDD in the communication, gross motor, fine motor, problem solving and social-personal areas varied between 1.1 and 2.6 % at 4 months, 0.6 and 2.3 % at 6 months and 0.4 and 3.6 % at 12 months by the Norwegian cut-off scores. The highest prevalence was found in the gross motor area in all three age groups, 2.6 % at 4 months, 2.3 % at 6 months and 3.6 % at 12 months. We also found that 1.8 % of infants with complete ASQ scores had a delay in more than one area at 4 months, 1.1 and 0.8 % at 6 and 12 months, respectively.

Table 3 shows the associations of gestational age < 37 weeks, gender, maternal education with developmental delay for each area and age groups. Gender was significantly associated with fine motor area (*p* = 0.029) and problem solving area (*p* =0.010) at 4 months and personal-social area at 6 months (*p* = 0.013), with a higher prevalence of SDD among boys. Gestational age of < 37 weeks was significantly associated with delay in the communication area (*p* = 0.001) at 4 months and the fine motor (*p* = 0.049) and personal-social area (*p* <0.001) at 6 months. Maternal education had no significant associations with the areas of the Ages and Stages Questionnaire in any age group.

**Table 3:** Association between gender, gestational age, maternal education and the area of ASQ

4 months	Communication			Gross motor			Fine motor			Problem solving			Personal- social		
	Prev^a^		*p*	Prev^a^		*p*	Prev^a^		*p*	Prev^a^		*p*	Prev^a^		*p*
Gender	1.2	1.6	0.538	3.4	2.0	0.138	2.9	1.1	0.029*	3.9	1.4	0.010*	1.5	0.9	0.331
Gest.age < 37 weeks	1.1	6.1	0.001*	2.3	6.1	0.058	1.7	3.0	0.437	2.0	4.5	0.168	1.1	1.5	0.787
Maternal education	1.5	1.5	0.953	2.7	2.8	0.954	1.0	2.6	0.061	2.2	3.0	0.414	1.0	1.4	0.561
6 months	Communication			Gross motor			Fine motor			Problem- solving			Personal- social		
	Prev^a^		*p*	Prev^a^		*p*	Prev^a^		*p*	Prev^a^		*p*	Prev^a^		*p*
Gender	0.9	0.4	0.312	1.9	2.7	0.395	2.1	1.3	0.344	0.9	1.1	0.643	2.4	0.6	0.013*
Gest. age < 37 weeks	0.7	0.0	0.520	2.4	1.7	0.733	1.6	5.1	0.049*	1.1	0.0	0.419	1.1	8.5	<0.001*
Maternal education	0.8	0.6	0.662	3.1	1.9	0.168	1.9	1.6	0.727	0.8	1.1	0.589	1.9	1.4	0.590
12 months	Communication			Gross motor			Fine motor			Problem- solving			Personal- social		
	Prev^a^		*p*	Prev^a^		*p*	Prev^a^		*p*	Prev^a^		*p*	Prev^a^		*p*
Gender	0.8	0.5	0.703	2.3	4.3	0.115	0.5	0.0	0.169	2.3	1.3	0.334	0.5	0.5	0.952
Gest. age < 37 weeks	0.7	0.0	0.578	3.1	7.1	0.151	0.3	0.0	0.726	1.9	2.4	0.825	0.6	0.0	0.619
Maternal education	0.9	0.6	0.626	2.2	3.8	0.268	0.4	0.2	0.769	0.4	0.2	0.538	1.8	1.9	0.826

^a^Prev = Prevalence(%), boys vs girls, ≥37 weeks of gestational age vs < 37 weeks gestational age, high vs low maternal education (higher education: had qualified from, or studied at the university or college).*Significant at *p* < 0.05

## Discussion

The aim of this study was to estimate the prevalence rates of SDD in a community sample of infants at 4, 6 and 12 months of age based on their ASQ scores in five developmental areas, as well as the associations of SDD with gender, prematurity (a gestational age of < 37 weeks) and maternal education. The results suggest that between 5.7 to 7.0 % of young infants between 4 and 12 months had SDD according to the Norwegian ASQ cut-off points, and between 10.3 to 12.3 % according to the US cut-off points. The majority of these had an indication of delays in one area only, most frequently in the gross motor area. Prematurity was significantly associated with SDD in the communication area at 4 months and fine motor and personal-social areas at 6 months. Significant associations were found between gender and the fine motor and problem solving areas at 4 months and the personal-social area at 6 months.

Previous studies have shown substantial variations in the prevalence of developmental delay. A number of methodological issues make it difficult to compare available prevalence rates, such as differences in case definition and criteria, type of measures used, variations in age and whether the studies report on low or high risk populations. Prevalences of developmental delay based on the National Health Interview Surveys (NHIS-CH), which is a parent completed questionnaire on development disability, reported that 15 % of US children between 3 and 17 years had a developmental disability [17]. The Health Intervention Survey (NHIS-D) on Disabilities reported that 3.4 % of all children had general developmental delays and 3.3 % had functional developmental delays among American children between 4 and 59 months [33]. A nationally representative longitudinal sample in the US showed that almost 13 % of the infants who were objectively measured by the Bayley Short Form-Research Edition at 9 and 24 month had developmental delays [34].

The results from the current study were based on a Norwegian version of the ASQ, and the data was gathered from well-baby clinics where almost the entire population of parents with young infants came regularly with their child for a developmental check-up. ASQ was implemented in all nine well-baby clinics in five municipalities with the intention of standardizing the general developmental monitoring and check-up by public health nurses, engaging parents as active partners and increasing the detection rate of infants at risk for SDD. Parents brought the completed ASQ form along to the 4, 6 and 12- month check-ups. To the best of our knowledge, few prevalence estimates exist of SDD based on parent-completed ASQ data collected in a primary care setting. Two studies yielded ASQ data collected from preventive health care clinics in the Netherlands. Prevalence rates for 4-year-old full-term children in the first study were 7.2 % for children with low sosio-economic status (SES), 4.8 % for intermediate and 2.8 % for high SES children [35]. The second study reported prevalence rates for full-term and moderate preterm children (43–49 months), at 4.2 % and 8.3 %, respectively [36]. The prevalence rates from these two studies are in line with the findings in the present study, which indicates prevalence rates between 5.7 and 7.0 % (Norwegian cut-off points, and between 10.3 and 13.3 % for US cut-off points). Other studies among younger children report higher rates of SDD measured with ASQ than our study [18, 19, 37–40]. A prevalence of 27 % was found in a well-child clinic among American children from 9 to 31 months [38], while another study reported a prevalence rate of 28.8 % among 9, 18 and 30 month old children who attended an ambulatory well-baby clinic in Chile [40]. However, the Norwegian ASQ normative sample reported prevalence rates of 10.3 % at 4 months, 11.8 % at 6 months and 11.6 % at 12 months [18], and a more recent Norwegian population-based study of 6 month old infants from the capital of Norway found that approximately every third infant obtained an ASQ score at or below the cut-off scores in at least one area according to the Norwegian and US recommended cut-off scores [19]. The Norwegian ASQ normative sample had a relatively small sample size in each age group, and unlike the present study, the participants in both the previous Norwegian studies received an invitation letter, completed the ASQ at home and returned it by mail to the researchers without any feedback. The prevalence rate found in the study by Alvik and Grøholt is unexpectedly high, especially when taking into consideration that infants with a birth weight below 2.5 kg and mothers with non-Scandinavian ethnicity were excluded from the study. In addition, none of the 14 pictograms in the ASQ 6-month questionnaire were included in the ASQ form that the parents were asked to complete at home. [19]. This might possibly have contributed to misinterpretations of the meaning of items and thus an incorrect response.

Developmental delay in one area is often found to be correlated with delay in other areas [41, 42], but being late in one isolated area only is associated with less risk for the child [43]. In our community sample, we found that only 1.8 % of the infants had SDD in more than one area at 4 months, 1.1 % at 6 months and 0.8 % at 12 months. The highest prevalence rate in the present study was found in the gross motor area during the first year of life, 2.6 % at 4 months, 2.3 % at 6 months and 3.6 % at 12 months. This was also the case in the Norwegian reference sample at 12 months (5.5 %) [18]. Still these gross motor prevalences are considerably lower than studies on young children from the US and other countries [26, 33, 39]. Motor development may differ in rate and sequence among infants and children from various cultural backgrounds [44]. It is also well recognized that the development of gross motor skills during early childhood is of paramount relevance for a child’s overall development [45], and a developmental delay in the ASQ motor area in early life has been found to predict later communication [41] and cognitive skills [42]. Several factors affect motor development among children, such as a child’s characteristics (e.g. gender, age, ethnicity and somatic conditions), child-rearing practices, parental/social expectations and the quality and quantity of stimulation provided in home [46]. For example, the caregivers’ attitude and encouragement toward an infant’s tummy time or floor time might be related to the child’s motor performance. Parents in Norway have 12 months leave and spend most of the day together with their infants, and the public health nurses encourage parents to stimulate their infant’s motor development in the first year of life. This may well have contributed to the relatively low prevalence rates in our sample.

Significant associations were found between gender and developmental delay in the fine motor and problem solving area at 4 months, and at the personal-social area at 6 months, with lower mean scores for boys in all areas. The finding that boys have a significantly higher rate of delay is in accordance with other studies of gender differences in preschoolers [28, 33, 47, 48]. On average, Richter and Janson showed that the developmental stage for girls in a Norwegian population was higher than for boys in all ASQ areas, except for gross motor function, in which no significant differences were detected [28]. The gender differences found in this study correspond with the results from previous research, therefore it seems preferable to develop norms for the Norwegian version of the ASQ separately for boys and girls in order to avoid false-positive classifications of boys in further assessments and interventions.

Premature birth (<37 gestation weeks) was associated with a delay in the communication area at 4 months, and the fine motor and personal-social area at 6 months. Developmental delays are common in preterm children and the risk increases with a decreasing geatation age (GA) [36, 49], which can be explained by the developmental stage of the central nervous system at birth [50]. Evidence from neuroscience shows that microstructural and neural connectivity processes are disturbed because of prematurity, and these disturbances may result in an atypical differentiation of neuronal pathways [51]. Premature birth was reflected by a delay in all five ASQ areas in the Norwegian study by Richter and Janson, although these negative consequences were seemingly more pronounced within the fine motor skills, problem solving skills and personal-social skills than the other areas [28]. Kerstjens and colleagues also found that both moderate and early preterm children measured with ASQ at 4 years of age had more frequent problems with fine motor, communication and personal-social functioning compared to their 4 year old peers born full-term [36].

Maternal education had no significant associations with the areas of the Ages and Stages Questionnaire in this study, in contrast to previous reported findings of the impact of maternal education upon child development [28, 31, 52]. There may be several explanations for why maternal education was not significantly related to infant development in this study. Firstly, this study was based on a Norwegian community sample, with a relatively high education level among the parents. Furthermore, our study was conducted on young infants between 4 and 12 months of age. In this early stage of development, biomedical factors may have a greater impact on development than the parents’ educational level. In addition, the Norwegian society also provides a highly stable and comprehensive social, financial and health care network that protects mother and babies to a high degree.

Prevalence estimates on SDD in young infants are scarce and a necessary first step in order to plan for early intervention. This study contributes to building a more comprehensive picture of young Norwegian infants’ developmental status. The sample is population-based with a relatively large sample size, and the ASQ is performed in a naturalistic setting in accordance with the recommended use of the instrument [26] . There were no exclusion criteria for participation in the study, but the families who did not want to participate differed from the participating parents in terms of having a lower educational level and a higher proportion of non-Scandinavian-speaking parents. This may have biased our results to some extent, but we did not find significant relationships between the mother’s education level and the child’s ASQ scores. There were no significant differences between the participating and non-participating children at the time of inclusion in the study. However, there was a reduction in the proportion of low birth weight infants with a completed ASQ from 4 to 12 months of age, which may have influenced the results and reduced the estimated prevalence of SDD. The Norwegian version of the ASQ was used and the Norwegian ASQ items are well translated and back-translated; thus, there is little probability of translation distortion [18]. It would have been preferable if a concurrent Norwegian validation of ASQ was available, but no such validation yet exists. Hence, the results of SDD among 4–12 month old infants in Norway must be interpreted with some caution.

## Conclusion

The current study contributes to a limited knowledge base regarding the prevalence of infants at risk for developmental delay. This large, representative regional population-based sample suggests a prevalence rate of SDD between 5.7 and 7.0 % among infants between 4 and 12 months of age based on the Norwegian cut-off points (10.3–12.3 % according to US cut-off points). During the first year of life, delays are most frequently reported within the motor area. Special attention should be paid to infants born prematurely and to boys, and separate norms for boys and girls should be considered for the ASQ.

## Additional file

Additional file 1:
**Norwegian (N.ref.)and US cut-off values at 4, 6 and 12 months Description of dataset- Shows the recommended cut-off scores in Norway and US at 4, 6 and 12 months.** (PDF 20 kb)

## Abbreviations

ASQ: Ages and Stages Questionnaires
N ref: the Norwegian reference sample
DD: Developmental delay
GA: Gestational age
GP: General practitioner
