# Supplementary material for: Prevalence of suspected developmental delays in early infancy: results from a regional population-based longitudinal study
Source: BMC Pediatr. 2015 Dec 17;15:215. doi: 10.1186/s12887-015-0528-z (PMC4683867; doi:10.1186/s12887-015-0528-z)
Supplement: Additional file 1: — Norwegian (N.ref.)and US cut-off values at 4, 6 and 12 months Description of dataset- Shows the recommended cut-off scores in Norway and US at 4, 6 and 12 months. (PDF 20 kb) [file 12887_2015_528_MOESM1_ESM.pdf]

Additional file 1

Norwegian (N ref.)and US cut-off values at 4, 6 and 12 months

	**4 months**		**6 months**		**12 months**	
	**N ref.**	**US**	**N ref.**	**US**	**N ref.**	**US**
	**cut-off**	**cut-off**	**cut-off**	**cut-off**	**cut-off**	**cut-off**
**Communication**	30	34.6	25	29.65	10	15.64
**Gross motor**	35	38.41	15	22.25	5	21.49
**Fine motor**	20	29.62	25	25.14	30	34.50
**Problem-solving**	30	34.98	30	27.72	25	27.32
**Personal- social**	25	33.16	20	25.34	15	21.73

Recommended Cut-off scores in the US (Mean – 2 SD) and in the Norwegian manual (primarily based on the 2 percentile) in the areas of the Ages and Stages Questionnaire
